# Empowering the Vehicular Network with RIS Technology: A State-of-the-Art Review

**DOI:** 10.3390/s24020337

**Published:** 2024-01-05

**Authors:** Farheen Naaz, Ali Nauman, Tahir Khurshaid, Sung-Won Kim

**Affiliations:** 1 Information and Communication Engineering Department, Yeungnam University, Gyeongsan 38541, Republic of Korea; farheenaaz@yu.ac.kr (F.N.); anauman@ynu.ac.kr (A.N.); 2 Department of Electrical Engineering, Yeungnam University, Gyeongsan 38541, Republic of Korea

**Keywords:** reconfigurable intelligent surface, vehicle-to-everything, NOMA, MEC, platooning, autonomous vehicles, intelligent transportation system, physical layer security (PLS)

## Abstract

Reconfigurable intelligent surfaces (RIS) are expected to bring about a revolutionary transformation in vehicular networks, thus paving the way for a future characterized by connected and automated vehicles (CAV). An RIS is a planar structure comprising many passive elements that can dynamically manipulate electromagnetic waves to enhance wireless communication by reflecting, refracting, and focusing signals in a programmable manner. RIS exhibits substantial potential for improving vehicle-to-everything (V2X) communication through various means, including coverage enhancement, interference mitigation, improving signal strength, and providing additional layers of privacy and security. This article presents a comprehensive survey that explores the emerging opportunities arising from the integration of RIS into vehicular networks. To examine the convergence of RIS and V2X communications, the survey adopted a holistic approach, thus highlighting the potential benefits and challenges of this combination. In this study, we examined several applications of RIS-aided V2X communication. Subsequently, we delve into the fundamental emerging technologies that are expected to empower vehicular networks, encompassing mobile edge computing (MEC), non-orthogonal multiple access (NOMA), millimeter-wave communication (mmWave), Artificial Intelligence (AI), and visible light communication (VLC). Finally, to stimulate further research in this domain, we emphasize noteworthy research challenges and potential avenues for future exploration.

## 1. Introduction

**Figure 1:**
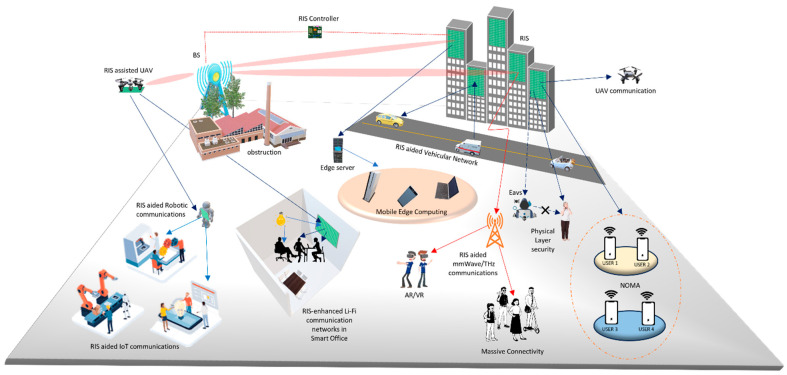
Emerging applications of RIS in wireless communication networks.

The transportation industry has made significant advancements with the introduction of 5G and beyond 5G (B5G) solutions, which have been specifically developed to enhance communication between vehicles and various elements of the transportation ecosystem. The emergence of V2X communications aims to enhance safety and convenience in daily travel, enabling advanced intelligent applications, such as intelligent transportation systems (ITS) and autonomous driving [1]. This advanced technology supports high-throughput and ultrareliable low-latency communications (URLLC), thereby improving traffic safety. Computational intelligence and real-time wireless communication play crucial roles in URLLC vehicular systems. Nevertheless, the impact of computational intelligence on ITS is significantly shaped by elements like the quantity and caliber of data, along with the control of disruptions in data transmission [2]. The Internet of Vehicles (IoV) transcends vehicular ad hoc networks (VANETs). This signifies a transition from considering vehicles as mere smartphones to recognizing them as intelligent and connected entities within a dynamic environment. Despite these advances, ITS research activities, products, and standardization continue to rely on WLAN and C-V2X for wireless connectivity in VANETs. Nonetheless, these technologies encounter a range of challenges, including amplified interference, extended communication delays, and diminished packet reception rates, particularly in situations with a high density of vehicles. Furthermore, as the quantity of self-driving cars continues to rise, the utilization of RF spectra in V2X applications is projected to expand. As a result, novel strategies are imperative to address these concerns and elevate the efficiency of V2X communications [3]. Several variables, including the signal-to-noise ratio (SNR) and the wireless channel’s fading effect, have an impact on the dependability of transmission channels in vehicular networks. Proposing the integration of dynamic, software-controlled RISs in real time is a strategy put forth to enhance the dependability of V2X communication. These specialized RIS units offer the possibility of enhancing the efficiency of CAVs by concurrently amplifying the strength of received signals and counteracting signal fading. These RIS units consist of metamaterial pixels with the capability to dynamically adjust the reflection coefficients and phase shifts of individual pixels. This allows real-time control over the angle of reflection, which is accomplished through software-based mechanisms [4]. The vision of emerging applications of RIS that will be discussed in this article is illustrated in Figure 1.

By integrating metasurfaces and employing efficient deep learning methodologies, it becomes possible to optimize the radio environments in conjunction with the operations of devices both at the source and the destination.

### 1.1. Current Research Status of RIS-Aided Vehicular Networks

Intelligent reflecting surface (IRSs), often referred to as RIS, represent an emerging technology with the capability to completely transform wireless communication networks [5]. Comprising numerous compact antennas, an RIS can be configured to redirect radio signals to pathways. This capability enhances signal potency, minimizes disturbances, and reinforces security measures. Significant amount of research has been conducted in the literature on RIS-aided wireless communication systems, including studies on diverse applications such as RIS-carried unmanned aerial vehicles [6], RIS-aided simultaneous wireless information and power transfer (SWIPT) [7], and RIS-assisted Massive MIMO [8]. The positioning of RIS units holds significant importance in defining their efficiency and influence on wireless communication systems. Through a comparison of metamaterial pixels, the RIS components possess the capability to adjust the reflection angle in real time using software. This is achieved by dynamically modifying the reflection coefficients and phase shifts of each pixel. RIS in millimeter-wave vehicular communications were investigated in [9,10]. In [11,12], the potential impact and benefits of reconfigurable metasurfaces for improving the safety of next-generation vehicles were discussed. Research has been conducted on physical layer security systems in relation to V2X communication aided by reconfigurable intelligent surfaces, as seen in studies referenced as [13,14].

### 1.2. Foregoing Work

Before detailing the contributions of this study, a discussion of the previous work is provided. The usability of LTE for supporting vehicular applications was investigated in [15,16,17,18]. In [16], the authors delivered a comprehensive guide outlining the 3GPP Release 16 5G NR V2X standard, emphasizing the sidelink, which holds utmost importance within the 5G NR V2X framework. A summary has been provided in [17] for the architecture and operational situations concerning LTE V2X. Moreover, the obstacles within current LTE networks to facilitate V2X communications are elucidated, including aspects like the physical layer configuration, synchronization, multimedia broadcast multicast services (MBMS), resource distribution, security, and an overview of recent strategies devised to address these hindrances. Various security and privacy elements throughout the system stack are taken into account by [19,20,21,22], in addition to road safety. Multiple security- and privacy-related shortcomings and inconsistencies across standards were identified in [19]. This involves exploring the possibilities of emerging security methods that utilize Artificial Intelligence techniques to achieve security goals. A summary of recently enabled use cases for 5G-V2X slicing and the associated security concerns is given, with particular attention to attacks involving cross-border network slicing (NS) provided by [21].

Some surveys and tutorials [23,24,25,26,27,28,29] introduced RISs and their variants. An overview of the RIS with a particular focus on hardware aspects is provided in reference [23]. A comprehensive technical discussion that can assist and stimulate future research on the modelling, analysis, design, optimization, and implementation of wireless networks aided by an IRS is provided in [24]. The functioning principles of RIS and the difficulties linked to fine-tuning RIS parameters for the purpose of boosting overall system performance are illustrated in the content [28]. Similarly, an extensive analysis of the integration of RIS with emerging communication technologies was presented in [29], specifically focusing on their application in realizing 6G wireless networks. Prior research has indicated that RISs can notably amplify the performance of the physical layer, encompassing aspects like wireless coverage, data rate, and energy efficiency. Nevertheless, the advancement of medium access control (MAC) mechanisms tailored for numerous users accessing channels facilitated by RISs is still at its initial phases. In [27], the authors focused on four common RIS-aided multiuser scenarios and placed particular emphasis on MAC schemes. In the context of multiuser communication systems aided by RIS, they presented and provided detailed explanations of centralized, distributed, and hybrid MAC architectures supported by Artificial Intelligence techniques.

The comparison of the related works and our article is summarized in Table 1. A comprehensive review discussing the integration of RIS in vehicular networks is presented in [11,30,31]. The highlighted focus on the potential uses of reconfigurable metasurfaces, particularly within vehicular settings was elaborated by [11]. This study focuses on two main use cases: (1) cooperative driving and (2) vulnerable road users (VRUs) detection. In [30], the primary focus was on the prospective transmission design of RIS-aided V2X communication. To fully realize the potential applications of RIS in supporting V2X communications, the authors extensively discussed the main challenges that must be addressed. In [31], RIS-assisted VANETs were discussed, and the author proposed that the optimization of RIS phase shifts should be performed in conjunction with resource management and vehicular scheduling. This joint optimization approach is essential for maximizing the benefits and effectiveness of RIS in V2X applications. The study in [32] delineate emerging possibilities facilitated by ISRE-based (intelligent surface-assisted radio environment) 6G vehicular HetNets (Heterogeneous Networks). Additionally, they showcase a case study that employs the contextual bandit approach to identify the optimal intelligent reflective surface (IRS) for ensuring secure communications.

### 1.3. Paper Contributions

In this study, we give a general introduction of RIS technology, covering its fundamental principles, architecture, and applications. Additionally, we explored the potential applications of RIS in various fields, such as B5G for V2X Communications, V2X communication subsets, and the potential of RIS for improving vehicular networks. This study’s main contribution is an outline of the key ideas and methods employed in RIS-based vehicular networks. The rest of this paper is structured as follows.

- Examine the advantages and limitations of RIS in vehicular networks: This study explores the advantages of RIS in improving vehicle coverage, capacity, and energy efficiency, as well as its limitations and challenges.
- A review of the existing literature on RIS-based vehicular networks: This paper discusses recent research on RIS in vehicular networks. The purpose of this review is to discuss the use of RIS in a variety of vehicular communication scenarios.
- By showcasing various use cases that highlight the advantages of integrating RISs with V2X communications, this paper examines the applications of RIS-assisted vehicular communications. It also discusses the potential effects of this framework on areas like coverage improvement, spectrum sharing, resource allocation, physical layer security (PLS), platooning, and autonomous vehicles.
- Based on our comprehensive review, we identified emerging technologies that enable the integration of RISs in various application scenarios within vehicles.
- Conversations regarding the unresolved research hurdles and forthcoming pathways for vehicular networks employing RIS are included. This dialogue encompasses subjects like enhancing the arrangement of RIS units, formulating proficient algorithms for controlling RIS functions, and merging RIS with other emerging technologies like 5G and self-driving vehicles.

**Figure 2:**
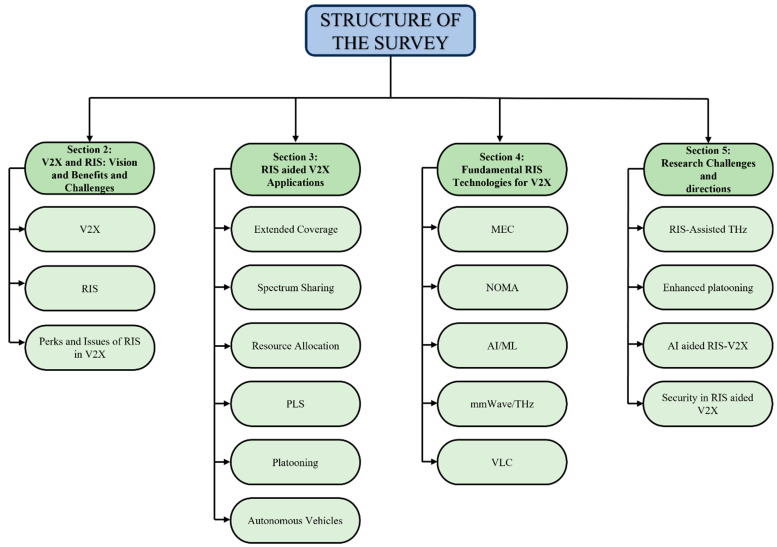
Organization of this article.

The subsequent sections of this paper are structured according to the layout depicted in Figure 2. Section 2 furnishes an elaborate account of the foundational aspects of V2X communication and RISs. Section 3 comprises several subsections that discuss important applications of V2X-assisted RIS. Section 4 focuses on emerging technologies and their impact on RIS and V2X, along with an overview of potential future research trends. In Section 5, a thorough exploration of unresolved obstacles and potential avenues for future research is provided. Concluding the review, Section 6 offers a summarized overview of the significant discoveries and insights extracted from the analysis. Table 2 encapsulates a compilation of essential acronyms referenced in this article.

## 2. V2X and RIS: Vision, Benefits and Challenges

### 2.1. Vehicle to Everything

In recent years, V2X communication has been the subject of extensive research in both academia and industry owing to its importance as a fundamental element of ITSs. Recently, the concept of the “Internet-enabled cars” has gained prominence, offering a new range of services to drivers through wireless communication. A pivotal element in the design of upcoming vehicles involves facilitating V2X communication, which unveils the complete capabilities of applications for CAVs. The dependability of these communication connections is greatly influenced by the SNR and the fading attributes of the utilized wireless channel.

**Figure 3:**
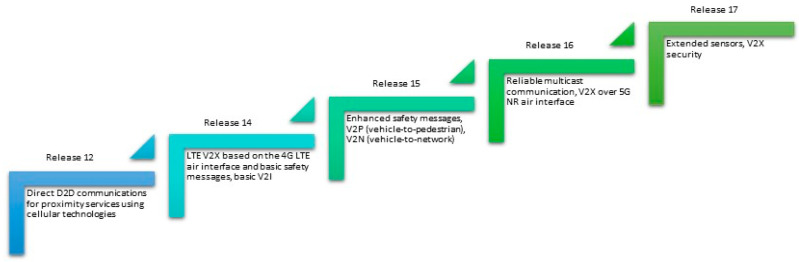
Summarizing the different releases of 3GPP and their V2X capabilities.

Direct device-to-device (D2D) communication for proximity services (ProSe) using cellular technologies occurred with the implementation of the 3rd Generation Partnership Project (3GPP) Release 12 (Rel. 12). Building on this foundation, 3GPP developed the LTE V2X, which became the initial cellular C-V2X standard based on the 4G long-term evolution (LTE) air interface. To address these requirements, 3GPP released the C-V2X specification in Releases 14 and 15. However, in Release 16, 3GPP introduced 5G New Radio (NR) technology with a focus on further enhancing the cellular V2X networking infrastructure. The journey towards 5G-Advanced V2X begins with the inception of 3GPP Rel.17, where the standardization process for NR-V2X initiates. This evolution commences with 3GPP Rel.16 and reaches its conclusion in 3GPP Rel.17. Currently, the 3GPP is actively engaged in developing Rel. 18, which aims to advance 5G capabilities. The primary focus of 3GPP Rel. 18 revolves around the seamless integration of Artificial Intelligence and machine learning (AI/ML) into the core radio access network (C-RAN), with the goal of enhancing overall network performance [33]. Figure 3 summarizes different releases of 3GPP and its capabilities in the context of V2X.

**Figure 4:**
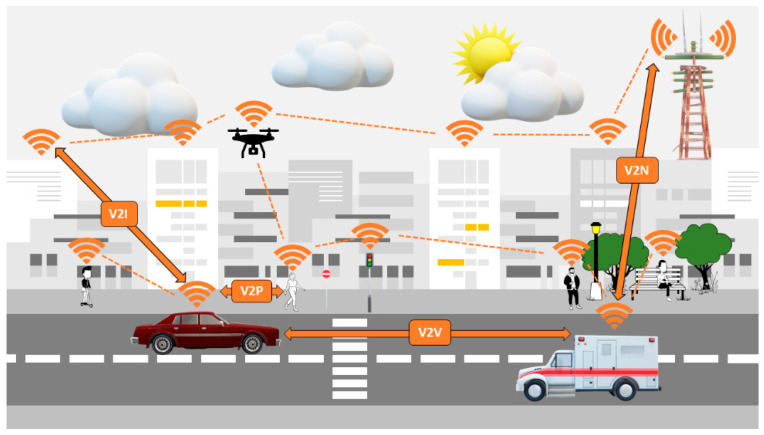
V2X communication.

The introduction of 5G NR is expected to become a game-changer in V2X communication, as it can complement or even replace the 802.11p standard. Building on the foundation of C-V2X, vehicular networks can achieve connectivity through various communication modes, such as vehicle-to-infrastructure (V2I), vehicle-to-pedestrian (V2P), and vehicle-to-vehicle (V2V) communications, as well as communication with cloud networks (V2N) [15]. As defined by 3GPP, the term V2X encompasses the communication among various entities, as shown in Figure 4, for both safety and non-safety applications.:

- Vehicle-to-Vehicle: According to the U.S. NHTSA, the implementation of a V2V system can reduce traffic accidents by at least 13%, resulting in 439,000 fewer crashes annually [34]. V2V communication involves direct wireless communication between two vehicles. This enables vehicles to exchange information regarding their location, speed, and travel direction. This information can contribute to enhancing safety by alerting drivers to potential dangers, such as imminent collisions. Additionally, V2V communication can be used to improve traffic flows by coordinating the vehicle movements [35].
- Vehicle-to-Infrastructure: It is one of the key elements advancing the communications market for infrastructure and vehicles. This enables communication between the infrastructure and the vehicles so that information about weather, road closures, and traffic conditions may be shared [36]. V2I devices collect data generated by moving vehicles and wirelessly transmit to the vehicle advisories on environmental, mobility, and safety conditions. State and local government organizations will probably build V2I infrastructure next to or integrated with current ITS hardware. Additionally, connectivity between the infrastructure and the vehicle offers a wealth of information for potential path optimization of the vehicle. V2I technologies also have the potential to enhance fuel economy and cut pollutants.
- Vehicle-to-Pedestrian: This communication involves direct wireless communication between a vehicle and a pedestrian or multiple pedestrians within proximity. This allows vehicles to exchange information with pedestrians such as their location and speed. This data have the potential to enhance safety by alerting drivers to potential risks, such as pedestrians crossing the road [37].
- Vehicle-to-Network: A V2N system is a connected vehicle concept and a component of the ITS. V2N refers to communication between vehicles, i.e., V2V and the surrounding network infrastructure, which can include roadside units, cellular networks, and other communication systems. To increase safety, lessen traffic congestion, and optimize travel routes, V2N technology enables cars to share data and communicate with one another and the infrastructure. Along with other uses, it can be used in autonomous vehicles. Smart cities can benefit from this technology as well because it can be used to improve traffic management and offer real-time information about the weather and road conditions. Emissions can be decreased, and efficiency increased with V2N technology.

V2X connections are made possible by two main Radio Access Technologies (RATs): (1) WLAN based and (2) cellular based [38].

#### 2.1.1. WLAN-Based V2X Communication

WLAN (Wireless Local Area Network) is a general term that includes various wireless communication standards, dedicated short-range communication (DSRC) is a specific subset of WLAN technology that stands as a wireless communication technology intentionally crafted for usage within vehicular settings. Vehicles can communicate with each other, the infrastructure, pedestrians, and other road users using DSRC-based V2X technology. It has been designed with the intention of fulfilling the demands of dynamic settings. Specifically, it addresses the need for characteristics such as high mobility support, low latency communication, and a considerable communication range to avoid accidents, safety-critical information such as the locations and speeds of other cars can be shared through this connection [39].

The DSRC uses the unlicensed 5.9 GHz spectrum, which is designated for ITS applications. Its primary feature is the ability to establish stable and dependable communication links. In addition to other communication protocols, DSRC makes use of IEEE 802.11p, a modified version of the Wi-Fi standard. DSRC-based V2X communication can be used in various applications, including:

- Collision avoidance: Vehicles can identify potential collision risks based on the data that have been processed. When a risk of a collision is detected, the system issues warnings to the driver and may even initiate autonomous actions to prevent the collision. Visual alerts on the dashboard, audio alarms, and haptic feedback through the seat or steering wheel are a few examples of these cautions.
- Traffic flow management: Vehicles equipped with DSRC communication devices exchange real-time traffic information with each other and with infrastructure elements. It can be used to communicate traffic information such as road closures, construction zones, traffic lights and road signs, to enhance traffic efficiency, reduce congestion, and improve overall road safety.
- Emergency alerts: When an emergency occurs, such as a crash, hazardous road conditions, or severe weather, authorized entities (such as emergency services, traffic management centers, or weather agencies) generate emergency alerts containing relevant information. Depending on the nature of the alert, the system might trigger visual, auditory, or haptic alerts to alert the driver about the emergency. DSRC-based V2X communication can be used for various types of emergency alerts, such as crash alerts, road hazard alerts, weather alerts and emergency vehicle alerts.

The DSRC-based V2X is a promising technology with the potential to improve road safety and efficiency. However, before DSRC-based V2X can be widely deployed, several challenges must be addressed, such as the following:

- Interoperability and Standardization: Developing and adhering to uniform communication protocols and standards is crucial for ensuring seamless communication between different vehicle manufacturers and infrastructure providers. Achieving global interoperability can be complex due to regional variations in standards. This challenge has been addressed through the development of standards for the DSRC-based V2X.
- Deployment: The deployment of DSRC infrastructure (roadside units, traffic lights, signs) requires significant investment and coordination with various stakeholders, including local governments, transportation agencies, and private companies. The cost of deploying a DSRC-based V2X infrastructure is challenging.
- Spectrum Availability and Allocation: The 5.9 GHz frequency band allocated for DSRC communication is limited and shared with other users, such as radar systems. Spectrum congestion can lead to interference and reduced communication reliability. Despite these challenges, the DSRC-based V2X is a promising technology with the potential to revolutionize transportation.

#### 2.1.2. Cellular-Based V2X Communication

Given the constraints inherent in DSRC, researchers are actively investigating other vehicular communication technologies that can serve as substitutes. LTE has emerged as a viable option for connected vehicles, owing to the extensive coverage provided by cellular systems and advancements in direct D2D communications. 3GPP is engaged in comprehensive research and specification efforts concerning LTE-based V2X communication. Within this context, a study initiative focusing on LTE-based V2X Services has gained approval from 3GPP. Notably, PC5-based V2V communication has been designated as the top priority within this study. The completion of the Radio Access Network (RAN) Feasibility study has encompassed the segment associated with PC5 transport specifically tailored for V2V services. Starting from 3GPP Release 12, LTE introduced D2D functionality, and a dedicated channel structure called a sidelink to facilitate direct communication. Recognizing the growing interest in the vehicular market, 3GPP began working on specific features of V2V communications, with significant enhancements included in Release 14 in September 2016 [40].

There are several advantages to using LTE for V2V communication. First, it leverages the same technology and hardware as cellular communications, allowing the utilization of existing protocols and infrastructure. This presents an advantage due to vehicles already being equipped with cellular interfaces, the ongoing enhancements of LTE specifications, and the extensive deployment of base stations.

Second, LTE provides orthogonal resources, enabling higher multiplexing and potentially enhancing reliability and capacity. However, this advantage comes with the tradeoff of increased complexity in terms of devices and protocols. Synchronization among devices has become crucial, and resource allocation, whether controlled by the network (sidelink mode 3) or performed autonomously (sidelink mode 4), is a critical aspect that requires careful consideration. We have demonstrated a comparison of DSRC and LTE based V2X technology in Table 3.

To narrow the performance disparity between DSRC and C-V2X and to facilitate additional operational modes with enhanced throughput, the IEEE established the IEEE 802.11 Next Generation V2X Study Group in March 2018. The initial 802.11p standard, crafted nearly two decades ago, inherited its PHY and MAC layers from 802.11a. However, subsequent technological advancements have given rise to 802.11n, 802.11ac, and the finalization stages of 802.11ax standardization. Recognizing the potential to harness advanced PHY and MAC techniques from these succeeding standards to improve upon 802.11p, the IEEE 802.11 Next Generation V2X Study Group was instituted.

In the genesis of IEEE 802.11p development, the primary focus centered on the creation of a vehicular communication standard aimed at bolstering vehicular safety, optimizing traffic management, and introducing value-added applications such as parking and vehicular diagnostics. After an initial feasibility study, the IEEE 802.11bd Task Group was established in January 2019. The key design objectives for 802.11bd encompass achieving, in at least one mode, twice the MAC throughput of 802.11p at relative velocities of up to 500 km/h, realizing at least one mode with twice the communication range of 802.11p, and integrating a form of vehicle positioning in conjunction with V2X communications [39].

### 2.2. Reconfigurable Intelligent Surface

RISs, also referred to as metasurfaces or IRS, consist of planar surfaces comprising an array of passive reflective components, each capable of independent manipulation to modify the phase and amplitude of incoming radio waves [41]. This allows RISs to be used to manipulate the propagation of radio waves in various ways, such as improving signal strength, coverage, and security. The objective of integrating RISs with next-generation multiuser technologies is to move the beamforming functionality from the traditional radio front ends of base stations, access points, and user terminals to the environment itself [26].

RIS-enabled smart radio settings can improve the intended signals and reduce interference by adjusting the phases and/or amplitudes of the incident signals. Through the incorporation and convergence of other promising technologies such as ultra-massive MIMO, terahertz communications, AI-driven wireless networks, and edge intelligence, a multitude of possibilities emerge for revolutionizing the architecture of wireless networks [42].

RISs are superior to conventional wireless communication technologies in several ways. First, they can combine signals with other pathways to improve the signal-to-interference-plus-noise (SINR) of the intended receivers as they are passive devices. In PLS networks, they can also reduce the signal strength of unwanted listeners. This passive nature of RISs improves their energy efficiency and enables them to operate in a more energy-efficient manner than traditional wireless devices [43]. The second benefit is that RISs are simple to install in a variety of indoor and outdoor locations. This characteristic renders them suitable for a diverse array of applications, including but not limited to smart cities, industrial automation, and mobile communication. Moreover, an IRS operates in the full-duplex (FD) mode, which means that it can simultaneously transmit and receive signals. Unlike traditional active relays such as half-duplex (HD) relays, the IRS does not experience antenna noise amplification or self-interference. Because HD relays often face challenges related to low spectral efficiency, this characteristic provides IRS with distinct advantages, whereas FD relays require complex techniques for self-interference cancellation. Finally, the RIS functions as an auxiliary device within wireless networks and can be seamlessly integrated into the existing systems, such as cellular or Wi-Fi networks. This integration was achieved without disruption and offered exceptional flexibility and compatibility. Consequently, IRS provides a highly adaptable solution that harmonizes with various wireless systems and enhances their overall performance [24].

Despite being a relatively recent innovation, RISs hold the capacity to completely transform the construction and implementation of wireless networks. A few potential uses of RISs are as outlined below.

- Enhanced signal strength: Metasurfaces can improve the strength of wireless signals by strategically reflecting on them, thereby increasing their power. This enhancement helps extend the coverage and reliability of wireless networks.
- Improved capacity: By optimizing the SNR, RISs boosts the capacity of wireless networks. They achieved this by reflecting signals to minimize interference, enabling more efficient utilization of the available spectrum.
- Reduced latency: It helps improve the reliability of wireless networks and reduce latency by effectively reflecting signals. By carefully manipulating signal reflections, RISs minimize signal loss, resulting in faster and more dependable communication.
- Enhanced security: RISs enhances the security of wireless networks. Manipulating signal reflection makes it challenging for potential attackers to intercept and decipher signals, thereby bolstering the overall security of the network.

**Figure 5:**
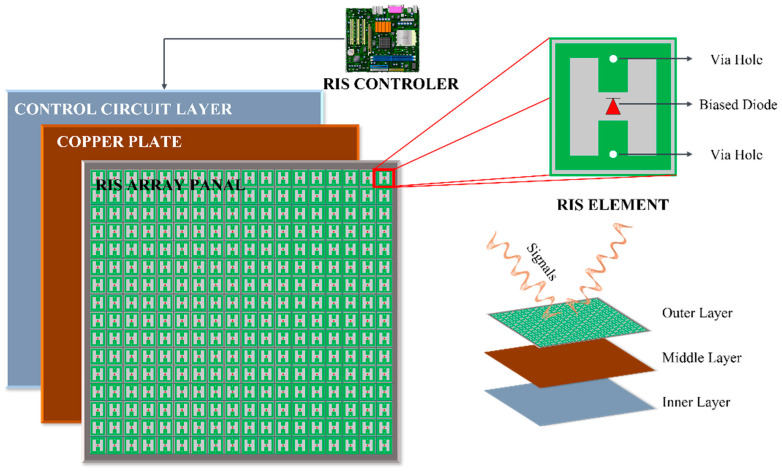
Architecture of an RIS.

As shown in Figure 5, the architecture of the RIS consists of three layers. The outer layer can be constructed using a single or multiple layers. In the design in [43], a three-layer planar surface was employed. This stratum engages directly with incoming signals, featuring multiple reflective components embedded on a dielectric substrate, thereby exerting a direct influence on the incoming signals. The intermediary stratum encompassed a copper panel to prevent any signal or energy leakage. The ultimate stratum encompassed a circuit board tasked with regulating the phase alterations of the RIS components. This fine-tuning is managed through an intelligent controller, such as a field-programmable gate array (FPGA). In the envisioned operational scenario, the base station (BS) calculates the optimal reflection coefficients of the RIS based on the channel state information (CSI). These coefficients are then transmitted to the RIS via a dedicated feedback link. Notably, CSI updates occur on a much longer timescale than the duration of the individual data symbols. Each individual reflective unit contains a positive-intrinsic-negative (PIN) diode seamlessly integrated into its structure, as depicted in Figure 5. By regulating the voltage applied via the biasing line, the PIN diode has the capability to alternate between active and inactive states, as demonstrated in the corresponding circuit diagram showcased in Figure 5. This switching capability allows the PIN diode to introduce a phase shift difference of π radians (180 degrees) [44]. Additional PIN diodes must be integrated into each element to increase the number of available phase-shift levels.

### 2.3. Benefits and Challenges in RIS-Aided V2X

In this section, we present a comprehensive analysis of RIS by exploring their various advantages and disadvantages in vehicular communication. In recent years, RIS has gained significant attention owing to its potential implications and applications in vehicular networks. By examining both positive and negative aspects, we aim to provide a balanced understanding of the topic and contribute to informed decision-making in this area. Table 4 comprises of benefits and challenges associated with RIS-aided vehicular networks.

#### 2.3.1. Benefits of RIS-Aided V2X Communications

- Range Extension: Non-line-of-sight (NLOS) environments, where direct line-of-sight (LOS) communication between the transmitter and receiver is obstructed, often pose challenges for long-range communication. The RIS can mitigate NLOS issues by redirecting and reflecting signals to reach the receiver through alternative paths. The RIS can improve the received signal strength and extend the communication range by optimizing the signal paths and mitigating multipath fading. In LOS scenarios with spatially sparse features, RISs can be utilized to create an artificially rich scattering environment. This emulation of a rich-scattering environment aims to enhance the channel condition number, which refers to the diversity and richness of the available propagation paths. Thus, an RIS can improve the spatial multiplexing capability of a communication system, allowing for the simultaneous transmission of multiple independent data streams [28].
- Energy Efficiency: RISs offer various avenues to enhance the energy efficiency of vehicular networks. An RIS can be used to focus radio waves on a specific direction, thereby reducing the amount of power required to transmit a signal. This is particularly important for V2X communication, in which vehicles must transmit short busty messages. They can also be used to amplify radio waves, which can improve the signal reception of vehicles in areas with poor signal quality. This can help reduce the amount of power that vehicles must use to receive V2X messages. Additionally, the RIS can be used to shorten the distance that radio waves need to travel, which can reduce the latency of V2X communication. This is important for applications such as collision avoidance in which vehicles must receive messages in real time. In pursuit of energy-efficient beamforming, the studies in [45,46] explored the incorporation of RIS.
- Increased Reliability: RISs can be used to improve the reliability of V2X communications by improving signal reception, reducing interference, increasing the range, and improving security. Improving the reliability and deployment of a metasurface in an appropriate location plays an important role. For, e.g., in urban areas, RIS can be deployed on roadside poles or buildings to improve the signal reception of vehicles traveling in urban areas and road intersections. In [4], architectural solutions to enhance the reliability of autonomous vehicular networks were proposed, which involved the deployment of real-time software-controlled RISs alongside roadways.
- Security: RIS offers several potential advantages for improving the security of V2X communications. By controlling the reflection of radio waves, an RIS can be used to create one-way or two-way channels that are only accessible to authorized vehicles. This can help prevent eavesdropping and spoofing attacks, as described in [43]. To calculate the SOP and validate them through verification processes, the authors in [47] derived closed-form expressions. Their research focused on understanding and evaluating the probability of information leakage in the presence of eavesdroppers in vehicular networks. The RIS can be used to improve the authentication of V2X messages and protect the privacy of communications.

#### 2.3.2. Challenges in RIS-Aided V2X Communication

- Double Fading: The presence of the “double-fading” effect poses a significant challenge that restricts the performance of intelligent surface-aided V2X communications. This phenomenon, often known as multiplicative fading, refers to the side effects introduced by RISs, where the signals received through the reflected links undergo twin-hop fading propagation [48]. This effect leads to significantly larger path losses compared with the direct link, making it challenging for passive RISs to achieve substantial capacity gains in many wireless environments, which characterizes the degradation of the signal strength over the transmission path. Addressing and mitigating this double-fading effect is crucial for enhancing the overall performance of communication systems.
- High Path Loss: RIS is a promising technology for improving wireless communication performance. However, in passive RIS configurations, where there are no active components to amplify or regenerate the signal, the path loss can be influenced by the reflective properties of the RIS elements. The specific design and configuration of the RIS, as well as the signal frequency and incident angles, can affect the efficiency of the signal reflection and the resulting path loss. In [49,50,51], some path loss models were proposed to improve the path loss.
- Cost and Infrastructure: Implementing RIS infrastructure over a wide area can be expensive and requires significant investment. The costs associated with manufacturing, installing, and maintaining many RIS elements throughout a vehicular network can pose practical and financial challenges to its deployment. Furthermore, the operational costs and energy consumption of RIS elements must be considered. In [52], a cost-effective, high-gain RIS with 2 bit capability was proposed. The RIS design utilizes a low-cost FR4 substrate and incorporates features such as the estimation of the signal arrival direction within the sub-6 GHz frequency band [53]. Although alternative types of switches may exist that can be utilized in RIS design, when considering cost-effectiveness, PIN diodes or varactor diodes are the most suitable options for designing low-cost RIS structures.
- Channel Estimation: Accurate channel estimation is crucial for optimizing the performance of RIS-aided V2X systems. However, in practical scenarios with varying channel conditions, high mobility, and multipath fading, obtaining a precise CSI for RIS elements can be challenging. The effectiveness of an RIS relies heavily on a timely and accurate channel estimation and adaptation, which can be limited to real-world vehicular environments. The performance of RIS-aided V2X systems is affected by Doppler-induced channel aging. This deteriorates system performance, particularly in terms of resource allocation. Successful resource allocation relies heavily on accurate and timely CSI. However, in highly variable channel scenarios, inherent CSI errors occur as the tracked information becomes outdated. Consequently, beamformed transmissions in such time-varying environments require robust resource allocation techniques specifically designed to mitigate the impact of stale CSI and ensure optimal performance [30].

## 3. Applications

**Figure 6:**
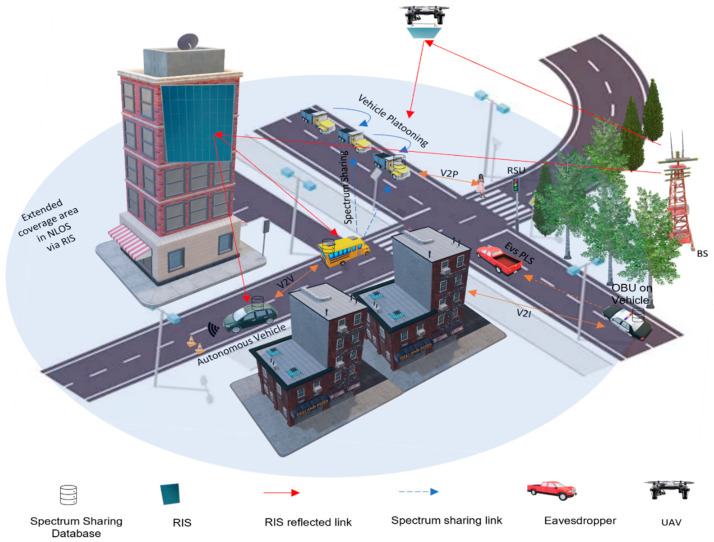
Applications of RIS-aided V2X.

Within this section, we embark on an extensive exploration and in-depth discussion of the burgeoning applications of RIS across a diverse array of critical V2X domains. These domains encompass extended coverage, resource allocation, spectrum sharing, bolstered physical layer security, autonomous vehicles, and platooning. For visual clarity, Figure 6 vividly depicts the myriad applications within the RIS-aided V2X environment.

### 3.1. Extended Coverage

The intelligent surface has the potential to improve various types of vehicular communication, including V2I, V2V, V2N, and V2S communications in 6G networks. Augmenting the propagation of multiple signal paths and prolonging the reach of transmission, particularly in elevated frequency spectrums like mmWave and terahertz (THz) [54]. An essential feature of an RIS lies in its capacity to intelligently adjust the signal direction toward the intended recipient. This feature allows the extension of wireless coverage and enhances system performance, particularly in non-line-of-sight communication scenarios, without consuming additional energy [55].

In [56], a downlink vehicular communication model was studied, in which communication occurs from a roadside unit (RSU) to vehicles via IRS-enabled buildings. This study considers situations in which the LOS of a vehicle is obstructed by other vehicles. The authors approximated the outage probability using series expansion and the central limit theorem. The outcomes of the simulations indicated that incorporating the IRS led to an enhancement in vehicle coverage and a decrease in the likelihood of outages. Similarly, in [57], the authors investigated an indirect transmission scenario in which RSUs communicated with vehicles in dark zones. In this investigation, a combined challenge involving resource scheduling and passive beamforming optimization for RSUs was formulated. The aim was to maximize the minimum average bit rate. To address this, Deep Reinforcement Learning (DRL) was harnessed for RSU wireless scheduling, while the passive beamforming optimization of the IRS was resolved through the utilization of block coordinate descent (BCD).

In [10] the initial findings demonstrated the potential for coverage enhancement in a vehicular application use case. To enhance mmWave networks, the authors demonstrated the anticipated benefits and challenges of deploying RIS in a joint mobility and network simulation framework. A novel adaptive localization scheme, the virtual line-of-sight (VLoS) technique, was presented in [58], by leveraging a beamforming protocol between an RIS and a mobile station (MS). To determine the location and orientation of the MS accurately, an interlaced scanning beam sweeping algorithm was introduced to identify the optimal beams. In a recently published article [59], a specific scenario is examined to demonstrate the efficacy of IRS in bolstering coverage and establishing LOS connectivity in challenging geographical settings. These settings, like tunnels or indoor environments, often suffer from a dearth of direct LOS, thereby leading to issues of insufficient coverage. An additional hurdle encountered in these ‘blind spots’, such as tunnels and indoor spaces, pertains to the absence of reliable access to the global navigation satellite system. The study showcased in this article serves to underscore how IRS technology can effectively address these challenges by harnessing its ability to manipulate wireless signal propagation.

### 3.2. Resource Allocation

V2X technology plays a pivotal role in various domains. It is a crucial enabler for enhancing road safety, improving traffic management, and enabling advanced mobility solutions. The V2X technology contributes to the development of ITS and paves the way for future advancements in autonomous driving, cooperative mobility, and smart city initiatives. Resource allocation is a critical aspect that directly affects system performance and efficiency. To address the challenges associated with vehicular communications effectively, numerous studies have introduced a range of resource allocation schemes and algorithms.

In [60], a hybrid architecture for V2V communication was proposed in which the cellular evolved NodeB (eNodeB) controlled the V2V links in an overlay scheme. The primary objective of cellular eNodeBs is to optimize resource allocation by selecting appropriate receiver vehicles to establish V2V links and allocating suitable channels in a way that minimizes the overall latency. A decentralized algorithm based on DRL was introduced in [61] to maximize the total capacity of V2I users and ensure latency and reliability requirements for V2V pairs. The simulation outcomes indicate that the DRL-based approach put forward exhibited superior performance compared to other decentralized benchmarks. Notably, the two-timescale federated DRL algorithm proved especially efficacious for recently initiated V2V connections, underscoring its exceptional effectiveness.

In [62], the joint power control and resource allocation challenges in D2D-based V2X communication were explored. The goal is to optimize the cumulative ergodic capacity of vehicular user equipment (VUEs) while accounting for imperfect channel state information (CSI), all the while ensuring that the minimum signal-to-interference-plus-noise ratio (SINR) prerequisites for cellular user equipment (CUEs) are met, along with complying with outage probability restrictions for VUEs. To address this problem, an improved Gale-Shapley algorithm is proposed to allocate spectrum resources effectively and solve the resource-allocation problem. The performance of C-V2X Mode 4, specifically in congested metropolitan areas, was proposed in a conference paper [63]. Furthermore, a resource allocation strategy was introduced, which results in a significant performance improvement. This improvement is particularly notable when accounting for the inherent half-duplex communication mechanism found in C-V2X Mode 4.

A RIS can assume a crucial function in the allocation of resources for vehicular communication. Research on resource allocation for RIS-aided V2X communication is limited. Pioneering work in this field can be found in [1], in which a resource allocation algorithm was proposed to enhance the quality of service (QoS) for RIS-aided V2X systems. The aim is to optimize the overall capacity of connections between vehicles and infrastructure while maintaining a minimum SINR for V2V links. This involves the simultaneous optimization of power distribution, IRS reflection coefficients, and spectrum allocation. These efforts collectively aim to attain the highest possible performance in communication enhanced by RIS in the V2X context. In the context of resource allocation in vehicular networks with RIS, several challenges are addressed in [30]. One significant challenge is the impact of Doppler-induced channel aging, which degrades the system performance over time. Additionally, the highly variable nature of the channels in V2X scenarios results in non-negligible errors in the CSI. This leads to stale information, which further exacerbates the problem. Consequently, robust resource-allocation techniques specifically designed for time-varying environments are crucial for beamformed transmission in RIS-aided V2X systems. These techniques must address the dynamic nature of the channels and mitigate the effects of CSI errors to ensure reliable and efficient communication.

### 3.3. Spectrum Sharing

With the escalating need for data transmission within vehicular networks, the constraint of having a restricted dedicated cellular spectrum presents a hurdle in fulfilling the demands of C-V2X users. Existing cellular spectral resources may become bottlenecks, thus restricting the capacity and performance of V2X systems [64]. The spectrum demand in V2X communication varies dynamically, depending on factors such as vehicle density and the specific applications involved. In scenarios with high vehicle density and large-capacity applications, wide-spectrum allocation is necessary to support seamless operations. To address this issue, spectrum allocation techniques are required, particularly when multiple communication standards must share the same spectrum band, as highlighted in [65]. To enhance spectrum efficiency, dynamic spectrum allocation strategies that consider the surrounding environment have been proposed to optimize spectrum utilization in V2X communication. The Federal Communications Commission (FCC) has designated 75 MHz of spectrum within the 5.9 GHz band for ITS [66].

Recently, the FCC released a notice of proposed rulemaking (NPRM), suggesting the possibility of allowing unlicensed devices to share a specific portion of this band while ensuring that another portion is exclusively reserved for safety-related traffic and not open for sharing [67]. A spectrum sharing scheme was developed in [68], which utilizes energy sensing to enable fair access to unlicensed channels for cellular V2X users. This scheme effectively reduced the occurrence of data transmission collisions. Even in complex scenarios, in which a spectrum-sharing system (SSS) coexists with multiple primary networks (PNs) or several secondary networks (SNs), an RIS can enhance both the spectral efficiency (SE) and energy efficiency (EE) [69]. Despite the challenges associated with the discretization of reflecting coefficients on the RIS, RIS-aided SSS holds immense potential for utilization in the field of vehicular communications. A novel three-stage algorithm called Joint Resource Allocation and Reconfigurable Intelligent Surface Optimization (JRARO) was introduced in [70]. This algorithm aims to maximize the sum capacity of the V2I communication by efficiently allocating resources and optimizing the configuration of the RIS. The proposed algorithm enables multiple V2I links to share spectrum resources while ensuring that various V2X communication requirements are satisfied. Similarly, the study in [71] concentrates on optimizing the cumulative capacity of V2I connections to facilitate high-rate content distribution. Simultaneously, it guarantees the dependability of V2V links for sharing information. This is achieved by enabling multiple V2V links to utilize the spectrum that has already been designated for V2I links. This study utilized simulation results to demonstrate the advantages of integrating RISs to enhance the QoS performance of vehicular communications.

### 3.4. PLS

For the successful deployment of V2X applications, it is essential to Ensure the security of vehicular communication. physical layer security (PLS) provides reliable solutions for combating eavesdropper attacks and complements cryptographic techniques to safeguard the integrity and confidentiality of communication. Enhancing road safety requires addressing a significant proportion of fatal car accidents attributed to human error. To achieve this, it is crucial to establish reliable and timely connectivity between autonomous and non/semi-autonomous vehicles. In this context, it is of crucial to ensure the confidentiality and security of the messages exchanged among vehicles [72]. V2X communication systems face various types of attacks, including jamming attacks (JA), Spoofing and Eavesdropping attacks (EvA). The implications of PLS attacks on V2X networks and the challenges they pose were discussed in [73]. In [74], solutions and [75] techniques were presented to address the aforementioned attacks and to enhance the secrecy of wireless communications within a V2X environment by employing PLS techniques. The risk factors associated with these threats to vehicular networks are categorized into three levels: high, medium, and low. In addition, an intelligent physical-layer security framework called intelligent V2X security (IV2XS) was introduced to enhance V2X communication security.

RIS has emerged as a prospective remedy for bolstering security at the physical layer in wireless networks. Incorporating RIS into the framework of physical layer security holds the promise of diminishing eavesdropping threats, augmenting signal confidentiality, and elevating the comprehensive security of wireless communication systems. A multiple-reflecting surface can also create multiple paths for V2X signals, making it harder for attackers to jam or intercept them. The study in [76] focuses on maximizing the secrecy rate in a wireless communication system by optimizing the system phase shifts at the RIS and the beam formers at the transmitters. This study proposes two efficient algorithms based on block coordinate descent (BCD) and minimization maximization (MM) techniques. These algorithms provide effective solutions for solving the non-convex optimization problem and optimizing the secrecy rate in the considered system.

To enhance the physical layer security of vehicular networks, several studies have focused on leveraging RIS. For instance, in [47], investigates the confidentiality performance of RIS-enhanced vehicular communication within two authentic settings. These settings encompass a dual-hop system and a receiver-based configuration, both operating in the presence of passive eavesdropping. Specifically, they analyzed SOP performance in two communication scenarios: V2V and V2I. In the presence of an eavesdropper, the study in [14] conducts a performance evaluation using two metrics: average secrecy capacity (ASC) and SOP. In this study, two vehicular system models were considered. The initial scenario centers on a V2V network, where the originating vehicle utilizes a RIS-enabled access point (AP) for transmitting. The subsequent configuration involves a VANET, in which an RIS-assisted relay is positioned on a structure. The results suggest that the integration of an RIS amplifies the secrecy capacity of a system. Furthermore, this research examines how different system parameters, such as source power, distance to the eavesdropper, and the quantity of RIS cells, impact the overall system’s effectiveness.

### 3.5. Autonomous Vehicle

Autonomous vehicles (Avs), also known as “advanced driver-assistance systems” (ADAS) or driverless cars, are vehicles capable of navigating and operating without human intervention. These vehicles are equipped with advanced technologies, including sensors, cameras, radar, lidar, and onboard computers, which enable them to perceive their environment, make decisions, and control their movements. The history of autonomous vehicles dates to the early 1900s, when inventors began experimenting with self-driving cars. However, it was not until the 1980s that significant progress was made in the development of autonomous vehicles. In the 1990s, there was renewed interest in autonomous vehicles as advances in computer vision and Artificial Intelligence made it possible to develop more sophisticated self-driving systems. In 1995, DriveAuto launched its first commercial self-driving car tour in Las Vegas. Google began its self-driving car project in 2004 and eventually became Waymo. In the 2010s, as companies such as Google, Uber, and Tesla began to develop self-driving cars, there was a surge in investment in autonomous vehicle technology. The Defense Advanced Research Projects Agency (DARPA) Urban Challenge, a competition to develop self-driving cars that could navigate urban environments, was held. In the 2020s, we began to observe the benefits of this investment. In 2017, Waymo began offering self-driving car rides in Phoenix, Arizona [77]. In 2023, the first commercial self-driving car fleet is expected to be launched in the selected cities.

Connected vehicles (CVs) are vehicles that rely on communication technologies to enable V2V and V2I communication. The testing and development of CVs have shown several potential applications, including cooperative adaptive cruise control, incident warning systems, traffic signal priority, emergency vehicles and transit priority, and smartphone-enabled pedestrian safety applications [78]. Recognizing the significant benefits in terms of safety, mobility, and environmental impact, the National Highway Traffic Safety Administration (NHTSA) has expressed considerations for mandating V2V communications. Vehicular technologies are advancing rapidly with new developments in connected vehicles, autonomous vehicles, and their integration in the form of CAV. The CAV can be understood in two ways [79]. The first refers to autonomous vehicles that are connected to other vehicles and/or infrastructure, capable of sensing their surroundings, and operate with minimal human intervention. Second, CAV encompasses a broader scope of technologies and applications focused on connected autonomous vehicles that collaborate with each other and the infrastructure. This collaboration aims to enhance road safety and efficiency by leveraging cooperative behavior and communication among vehicles, thereby surpassing the capabilities of autonomous vehicles operating independently.

The RIS could be a key technology in the future of autonomous vehicles. One application is enhancing the accuracy of Light Detection and Ranging (LiDAR) sensors, which are instrumental in creating a precise 3D map of the surroundings of a vehicle. RISs can improve the sensor performance by optimizing the propagation of LiDAR signals, thus enabling the vehicle to navigate safely and avoid obstacles in complex environments. Another application is to extend the range of radar sensors responsible for detecting nearby vehicles and objects. Through intelligent manipulation of radar signals, RISs can expand the sensing range, thus aid vehicles in collision avoidance, and maintain a safe distance from other vehicles. The study in [4] presents an architectural solution to enhance the reliability of autonomous vehicle networks. The suggested approach involves installing RIS units along the edges of roadways. This investigation centers on a scenario where a vehicle is traveling along a highway, utilizing cell-free mobile communication. In this setup, N RIS units are positioned perpendicular to the road. The primary objective is to ascertain the most advantageous placements for RIS units to optimize the average received power along the highway, thereby augmenting the connectivity for autonomous vehicles. The optimization procedure is executed individually for the focusing and beamforming modes of the RIS. To pinpoint the optimal positions, the optimum RIS positioning algorithm is employed, yielding positions of {0, 500} meters for the focusing mode and {123, 377} meters for the beamforming mode. The outcomes indicate that the incorporation of RIS elevates the power levels along the road, with the focusing mode surpassing the beamforming mode, as anticipated. A new RIS-assisted mmWave MIMO channel model was proposed to address the challenge of blockage awareness in autonomous vehicles in [80]. To evaluate the localization error probability, the model uses a channel-covariance splitting method and double-step iterative algorithm. This model can improve the localization accuracy and mitigate the blockage impact in mmWave communication channels, as shown by the simulations. The results of this research carry significance for the prospective developments in autonomous vehicles and mmWave communications. A recently presented conference paper [81] at the IEEE Vehicular Networking Conference (VNC) explored how RIS can be used to improve the localization accuracy of connected autonomous vehicles in millimeter-wave MIMO systems. By integrating the RIS and hybrid beamforming techniques, this study aims to enhance the localization performance of these vehicles.

### 3.6. Platooning

The utilization of platoon-based driving patterns offers several benefits, such as increased road capacity, improved fuel efficiency, enhanced comfort during road trips, and reduced energy consumption [82]. Platooning involves vehicles traveling together at synchronized velocities and maintaining a small distance between them, resulting in decreased aerodynamic drag and improved road capacity [15]. Effective management can enhance traffic safety and capacity by leveraging proximity and coordinated driving within a platoon. V2X communication technology plays a crucial role in facilitating platoon operations. Within each platoon, vehicles can exchange driving information and collaborate via V2X communication. This enables timely transmission of safety-critical information, including emergency alerts and traffic conditions, thereby enhancing the overall traffic safety [83]. However, cooperation among platoons is essential to ensure an optimal performance [17]. Without coordination, interference between adjacent platoons may become uncontrollable, and maintaining the QoS for intra platoon communication could be challenging.

Cooperative vehicular platooning (Co-VP) holds immense promise for enhancing road safety by reducing human intervention during driving. This concept involves autonomous vehicles working together in flexible platoons to enhance driving efficiency and safety. Recent research, as described in [84], has delved into advancements in Co-VP and focused on controlling strategies for vehicles, infrastructure communication, and cybersecurity. In [85], the study focused on investigating platoon cooperation in a multilane platoon scenario within a C-V2X network. The scenario comprised both cooperative and non-cooperative platoons. The objective is to analyze and understand the dynamics and challenges associated with platoon cooperation in such a network environment. This study explores the interactions and behaviors of cooperative platoons as well as the impact of non-cooperative vehicles on the overall system performance. Effective coordination in platooning relies on the reliable and periodic wireless exchange of control messages. However, in challenging propagation scenarios such as dense urban areas, coordination can be hindered, potentially resulting in undesirable vehicle behaviors. To overcome the challenges posed by challenging propagation scenarios in platooning, the use of RISs has emerged as a promising solution for enhancing coverage. The use of RISs in V2X communication, specifically in platooning, is a relatively new research area. As with any emerging technology, the number of studies conducted in this context is limited.

To the best of our knowledge, only two studies have explored the application of RISs in platooning. The authors in [86] have developed a simulator using the OMNeT++ framework, thus incorporating the PLEXE and Veins frameworks. Their simulation tool concentrated on assessing the effectiveness of RIS-assisted self-driving functionalities, giving specific attention to the integration of RISs within the mmWave spectrum. By surmounting obstructions due to line-of-sight issues and furnishing substantial bandwidth for sensor data, RISs present potential resolutions for collaborative driving circumstances, including platooning. The simulator accounted for vehicle movement, physical layer signal propagation, coding for RIS functionality, and networking protocols, thus enabling comprehensive and realistic assessments. The authors highlighted the importance of RIS-assisted mmWave links in extending the communication range, particularly in critical areas such as intersections where traditional communication may fall short. Their work contributed to the understanding of RIS-assisted communication in cooperative driving and provided a valuable tool for assessing the potential benefits of RIS technology in autonomous driving applications. Researchers in [87] explore the integration of RISs within vehicle platoons working alongside a base station (BS) to enable high-precision location tracking. The utilization of RISs introduces structured sparsity, which, when combined with the inherently sparse LoS channels of the BS, enhances group sparsity. To address this issue, researchers developed a hierarchical framework called diverse dynamic layer-structured sparsity (DiLuS). This framework was designed to capture diverse sparsity patterns while considering the spatiotemporal correlations among vehicles. By employing DiLuS, researchers have aimed to optimize the utilization of RISs in vehicle platooning scenarios and improve the overall efficiency of high-precision location tracking.

To provide a comprehensive overview of the technical aspects covered in each reference work, we present a compilation of the diverse applications of RIS-aided V2X in Table 5. This approach offers enhanced insights into the specifics of each application.

## 4. Emerging Technologies

**Figure 7:**
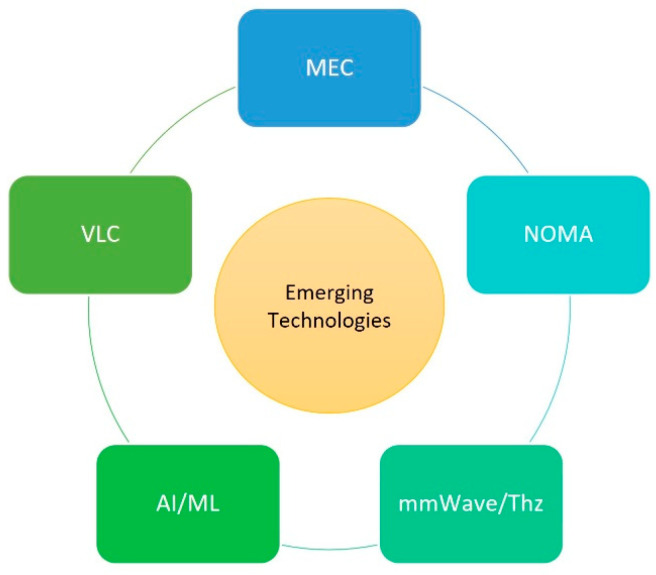
Emerging technologies.

In this section, our focus shifts towards the exploration of forthcoming technologies that are anticipated to pave the way for future RIS integrated vehicular networks and applications. Specifically, we delve into technologies that directly underpin RIS-enhanced vehicular networks. This includes an in-depth examination of MEC, NOMA, mmWave communication, AI, and VLC or Li-Fi as shown in Figure 7.

### 4.1. Mobile Edge Computing

MEC is a rising technology that holds a pivotal position in empowering functionalities that surpass the capabilities of vehicular networks in the 5G and 6G realms. With the advent of these new generations of cellular networks, latency requirements for connected cars have become extremely stringent, requiring response times within the range of 1 millisecond (ms) range to support a wide array of use cases. MEC has been identified as the optimal solution for addressing these ultralow-latency scenarios [88]. MEC achieves this by hosting applications in proximity to users in the edge cloud, thereby enabling the shortest possible path between the applications and end users. This is in contrast to traditional cloud computing, where applications are hosted in centralized datacenters that are often located far from the end users. Vehicular networks, with their demanding mobile applications, require efficient solutions to address performance challenges, particularly in terms of communication and computation delays. Mobile cloud computing (MCC) has emerged to address the QoS requirements of vehicle-dedicated applications, leading to the concept of vehicular cloud computing (VCC). Although the MCC offers promising advancements in mobility, the issue of time management persists. To overcome this problem, edge computing was introduced, which deploys servers closer to the data-generating equipment to achieve lower latencies and faster packet transmission and processing [89]. The integration of edge computing in VCC aims to optimize the performance of vehicular networks and enhance the overall user experience.

RIS are used in MEC networks to enhance the wireless communication performance. An RIS can passively reflect incoming signals, making it highly advantageous for MEC applications. Vehicular edge computing (VEC) has been suggested for intelligent transportation purposes, including tasks like monitoring road safety, facilitating autonomous driving, and preventing collisions. Through the dense placement of RIS within VEC networks and the adept modulation of phase shifts and amplitudes in these reflective components, it becomes feasible to dynamically manage and configure the wireless setting and network connection to attain performance optimization.

The researchers in [90] proposed a scheme that combined the RIS with edge computing to minimize the overall latency involved in offloading tasks. This scheme incorporates an actor-critic DRL algorithm. The problem of minimizing RIS configuration, computation resource, and offloading partition is formulated as a DRL problem, where the system state, action, and reward are defined. The state in the system comprises five components: $D_{k}$, $C_{k}$, $f_{k}^{1}$, $F^{s}$ and Θ. These components are assembled by the base station (BS) and transmitted to the DRL agent as the state representation. The action taken in the system consists of four parts, denoted as A = ($x_{k},f_{k}^{s},\beta_{n},\theta_{n})\;\;$, where $x_{k}$ represents the offloading variable, and $\beta_{n}$ and $\theta_{n}$ represent the RIS reflection coefficients and phase shifts, respectively. Based on the current state and action, the DRL agent interacts with the environment and receives a reward. The communication between the BS and mobile devices is facilitated by a single RIS consisting of N passive reflecting elements. The task is represented as ($D_{k}$, $C_{k}$), where $D_{k}$ denotes the data size of task k and $C_{k}$ represents the required computation resource for processing a unit bit. tasks were divided into two parts: one part is processed locally (1 − $x_{k}$), while the other part is offloaded to the edge server for processing ($x_{k}$).

(1) $$R^{imm}\left(s\left(t\right),a\left(t\right)\right)=-\sum_{k\epsilon K}max\{D_{k}C_{k}\frac{1-x_{k}}{f_{k}^{1}},D_{k}C_{k}\frac{x_{k}}{f_{k}^{s}}+D_{k}\frac{x_{k}}{R_{k}(g_{k})}$$

$$s.t.\sum_{k\epsilon K}f_{k}^{s}\leq F^{s},0\leq f_{k}^{s}\leq F^{s},k\epsilon K$$

$$x_{k},\beta_{n}\epsilon \left[0,1\right],k\epsilon K,n\epsilon N$$

$$0\leq \theta_{n}\leq 2\pi ,n\epsilon N$$

The variables $f_{k}^{s}$ and $F^{s},$ represent the computation resource allocated by the BS to task k and the total computation resource available at the BS, respectively. Numerical results obtained from this study demonstrate the efficacy of the RIS-aided scheme in enhancing the wireless communication data rate and reducing task execution latency.

The article [91] focuses on the study of MEC-enabled vehicular networks, leveraging the assistance of the IRS to enhance the computing performance of the system. To overcome the constraints arising from the presence of IRS links and MEC processors, the optimization of task scheduling was achieved through an evaluation of processor resource allocation and policies for offloading. In future vehicular networks, the integration of IRSs and MECs is anticipated to play a significant role in improving overall network capabilities.

### 4.2. NOMA for V2X-RIS

Owing to the increasing need for user connectivity, traditional orthogonal multiple access (OMA) technology has become inadequate for meeting user communication requirements [92]. NOMA has gained significant attention as a potential solution to next-generation multiple-access technologies [93]. Specifically, power-domain NOMA has emerged as a prominent approach that enables the allocation of the same frequency, time, or code to multiple users [94].

Considering the potential benefits of RIS, such as efficient signal reflection in full-duplex (FD) mode and low energy consumption, it is advisable to explore the incorporation of IRS into NOMA networks. This integration has the potential to improve both spectrum and energy efficiencies, thereby meeting the diverse requirements of 6G networks. To achieve spectral multiplexing, this technology utilizes successive interference cancellation (SIC) receivers at the weaker user ends. Although implementing NOMA may introduce additional complexity to the system, costs decrease as industrial technology advances [95]. In [96] the authors stated that both LTE-V2X and 5G-NR-V2X, which utilize OFDMA for multiple access, may encounter similar issues. However, with the rapid expansion of communication sensors and connected vehicles, extensive research has been conducted in recent years to address this challenge by exploring NOMA techniques. These NOMA approaches, including the power-domain and code-domain NOMA, can be applied to hybrid RF-VLC-based V2X communication systems to support a large number of simultaneous communication links.

In [97], an assessment is conducted on the effectiveness of networks employing RIS in the context of two-way non-orthogonal multiple access (TW-NOMA). Within this configuration, a user pair participates in information exchange through the employment of an RIS. The aim was to scrutinize the system’s throughput and energy efficiency within RIS-TW-NOMA networks, considering both the delay-constrained and delay-tolerant transmission modes. This analysis aims to provide insights into the effectiveness of the RIS technology in enhancing the performance of TW-NOMA networks by considering different transmission requirements. A novel approach called simultaneously transmitting/refracting and reflecting a reconfigurable intelligent surface (STAR-RIS) NOMA was proposed in [98] to achieve comprehensive coverage in a given area. In this study, approximate expressions for the outage probability of user n using either interuser power successive interference cancellation (ipSIC) or partial successive interference cancellation (pSIC) are derived, as well as for user m in the presence of Rician fading channels. Within this arrangement, the base station emits initial signals, which subsequently undergo reflection and transmission towards users situated in proximity as well as those at a greater distance. The derived expressions are used to analyze the outage performance of the system and understand the impact of different fading conditions on user reliability and system efficiency.

Considering the assumptions mentioned earlier, the identification of SNRs for users n and m within the framework of STAR-RIS-NOMA can be represented as follows,

(2) $$\gamma_{n}^{OMA}={\left|h_{sn}+h_{rn}^{H}\Theta_{R}h_{sr}\right|}^{2}pa_{n},$$

$$\mathrm{And}\;\gamma_{m}^{OMA}={\left|h_{rm}^{H}\Theta_{T}h_{sr}\right|}^{2}pa_{m}$$

The application of NOMA to vehicular networks has certain limitations, with channel loss being a significant concern. To address this issue, a NOMA-based Internet of Vehicles network assisted by RIS was proposed in [99]. This study explored two power allocation schemes aimed at mitigating the impact of channel loss. Specifically, power allocation schemes were designed to optimize the performance of different configurations of the reflecting elements within the RIS. This research delved into the balance between the quantity of reflective elements integrated into the reconfigurable intelligent surface (RIS) and the attenuation experienced by the signal paths. As a result, distinct elements were pinpointed that can proficiently cater to users positioned either in proximity or at extended distances. By meticulously accounting for these variables, the suggested strategy strives to enhance the comprehensive efficiency and dependability of NOMA based IoV networks amidst channel losses.

### 4.3. mmWave and THz

In the application section, advanced use cases for V2X communication are discussed, including vehicle platooning, extended coverage, and automated driving. These use cases require the exchange of larger messages through V2X to support “AI drivers,” which involve sharing raw sensor data, vehicles’ intention data, coordination, and confirmation of future maneuvers. Enhanced use cases have more stringent requirements, such as a data rate, latency, and reliability of 1000 Mbps, 3 ms, and 99.999%, respectively, for extended sensors, enabling cooperative perception for AI drivers. Recently, there has been a proposal to utilize mmWave technology to enable the sharing of raw sensor data in V2X. The key advantage of mmWave frequencies is their high bandwidth, which allows potentially high data rates [100]. For local area networking, mmWave was implemented in IEEE 802.11ad, thus offering a peak data rate of 6.75 Gbps. Additionally, it has been incorporated into 5G New Radio (NR) with a peak data rate of 20 Gbps. The integration of mmWave communication is expected to play a critical role in supporting advanced use cases of automated driving [101]. High-resolution real-time maps, known as dynamic High definition(HD) 3D maps, are essential for safe navigation of vehicles in automated driving systems [102]. LiDAR sensors are commonly utilized to generate these maps and monitor a vehicle’s surroundings. A LiDAR sensor enables the creation of a high-resolution and real-time point cloud that provides detailed information regarding the environment [103]. In densely populated urban areas, a vehicle’s view of the HD map can be blocked by other vehicles, surrounding buildings, and cars parked on the street, as signal propagation in mmWave communication is susceptible to the influence of physical obstructions.

To address the problem of direct link blockages caused by static or dynamic obstacles, an RIS was deployed to redirect mmWave signals to vehicular users. In [104], a risk-aware learning-based framework was proposed using the DRL approach. The framework aims to enhance road safety, which is the primary objective of ITSs. This is achieved by dynamically manipulating the propagated wireless signals to minimize the impact of link blockage. To ensure a seamless and uninterrupted connection, it is proposed that an RIS comprising N reflecting elements be positioned on a building. The RIS is designed to facilitate LoS links for vehicles traversing areas affected by blockages, thereby guaranteeing a continuous LoS connection between the BS and all vehicular users. To optimize coverage in vehicular networks a simulation framework called Lightweight ICT-centric Mobility Simulation (LIMoSim) was developed to effectively explore the possibilities of utilizing RIS [10]. This study conducted a coverage analysis using a three-step approach. First, only the LOS paths from a central base station were considered. In addition, the impact of deploying RISs on coverage enhancement was evaluated. Finally, to account for any misalignment between the RIS-reflected paths and vehicles, the simulation incorporates imprecise vehicle positions. The initial findings demonstrate the potential of RIS deployment to enhance coverage for a specific vehicular application use case. THz communications have demonstrated significant potential owing to their promising performance in short-range LOS scenarios. Consequently, the utilization of reflective RIS in mmWave and THz communications holds promise as a viable solution for overcoming the obstacles mentioned above in vehicular networks.

### 4.4. Artificial Intelligence

AI has become increasingly important in various engineering research fields owing to the advancements in computational technologies and hardware. In autonomous driving, AI plays a critical role in enabling essential human-like driving features. V2X technology can significantly enhance the safety and efficiency of autonomous vehicles [105]. Moreover, it has been acknowledged that integrating AI into RIS communications can significantly enhance reconfiguration capabilities and improve robustness in complex transmission environments [106]. The utilization of intelligent RIS in V2X applications enables power allocation, low-latency transmission, resource allocation, and autonomous decision-making. Machine Learning (ML) constitutes a subset of AI centered on the creation of algorithms and models that empower computers to acquire knowledge and formulate forecasts or choices devoid of direct programming instructions. ML algorithms can be categorized into supervised (e.g., linear regression, logistic regression, neural networks, SVM, Naive Bayes), unsupervised (e.g., k-means clustering, GMM, PCA), semi-supervised, and reinforcement learning (e.g., Monte Carlo, Q-learning, SARSA, PPO), each serving various applications such as predictive analytics, classification, sentiment analysis, computer vision, anomaly detection, recommendation engines, and industrial automation [107]. Numerous studies have been conducted on the implementation of machine learning in RIS-assisted V2X communication. In [108], the authors addressed the power allocation issue in the V2X environment by formulating it as the Weighted Minimum Mean Square Error (WMMSE) problem. In [109], the authors introduced a novel channel estimation method for an IRS-assisted B5G/6G network utilizing supervised learning based on Convolutional Neural Networks (CNNs). They considered the transmission power of the signal and aimed to minimize the power consumption. The experimental findings demonstrate that the proposed CNN-based channel estimation technique enhances the estimated mean squared error in the IRS, thus surpassing the performance of the baseline approach. The framework proposed in [110] introduces a hybrid vehicular communication structure that combines an IRS and DSRC cellular technology. A communication framework strengthened by AI has been introduced to VANETs with the aim of attaining energy efficiency, elevated data rates, and swift transmission with minimal delay. This framework encompasses a scheme for network-resource control and allocation based on DRL. This scheme proficiently governs variables such as vehicle transmission power, IRS reflection phase shift, and base station (BS) detection matrix, thereby facilitating energy-conscious communication within VANETs. The study in [111] proposed a control scheme called deep-learning-based IRS Phase Shift and Power Allocation Control (D-PPC), which utilizes deep learning techniques to optimize the phase shift and power allocation in an IRS. The objective of D-PPC is to minimize the transmission latency while ensuring QoS and meeting the reliability and latency requirements of diverse V2X services.

### 4.5. Light Fidelity or Visible Light Communications

Li-Fi or VLC is a wireless communication technology that uses visible light as the medium to transmit data. It is a promising alternative to traditional wireless technologies, such as Wi-Fi and Bluetooth, because it offers several advantages, including higher data rates, lower latency, more security, and easier deployment. Li-Fi can achieve data rates of up to 224 Gbps, which is much higher than the maximum data rate of Wi-Fi (6.7 Gbps). It also has a latency of less than 1 s, which is significantly lower than that of Wi-Fi (10 s). In addition, Li-Fi is more secure than Wi-Fi because it is difficult for eavesdroppers to intercept light signals. Finally, Li-Fi can be deployed more easily than Wi-Fi because it does not require the installation of a new infrastructure. VLC has evolved into a well-established communication technology, which is primarily suitable for indoor applications. It provides advantages, such as cost-effective deployment, exceptionally high data rates, and operation within unlicensed spectrum bands. The application of VLC, specifically Vehicular VLC (V-VLC), in outdoor environments is of particular interest. VLC serves as a valuable complement to Radio Frequency (RF)-based communications and offers significant benefits for automotive applications. V-VLCs can leverage abundant spectral resources and readily available LED-based lighting systems in modern vehicles to enhance their communication capabilities [112]. However, maintaining a LOS link and ensuring transceiver alignment between vehicles in highly mobile environments pose challenges. To address this issue, the authors of [113] suggested deploying RIS arrays to facilitate communication between vehicles that are moving parallel to each other. Because VLC transceivers have a limited field of view (FOV), maintaining alignment between two vehicles traveling beside each other becomes difficult. In [3], the authors suggested practical deployment strategies, specifically, a hybrid approach combining vehicular visible light communication (V-VLC) and vehicular-radio frequency (V-RF) with a relay. They also proposed incorporating RIS technology to enhance V-RF solutions. The goal was to amplify the communication coverage for V2V interactions within urban settings. A stochastic geometry-oriented analytical framework was employed to evaluate the effectiveness of our suggested approaches in relation to outage probability, throughput, and the rate of delay outage (DOR). The study in [114] introduces a VLC communication infrastructure designed specifically for vehicles, considering the unique characteristics of road tunnels, while researchers in [115] explore how RIS technology can effectively reduce the blocking probability (BP) within tunnels and mitigate signal blockage issues.

With the objective of concluding this endeavor, we encapsulate the emerging technologies of RIS alongside their pivotal attributes and significant employment scenarios within vehicular networks. These summative details are presented comprehensively in Table 6.

## 5. Research Challenges and Future Direction

In this section, we outline compelling avenues for future research that hold immense significance in unleashing the complete potential of RISs in vehicular networks.

### 5.1. RIS-Assisted Terahertz (THz) Communication

THz communication, which is known for its ultrawide bandwidth, has emerged as a compelling candidate for V2X communication owing to its impressive capabilities. However, THz signals suffer from significant signal attenuation and communication interruptions owing to their ultrahigh-frequency nature. This limitation severely restricts the practical deployment and range of THz communication systems. Nevertheless, RISs have emerged as a potential solution for addressing these drawbacks. Strategic deployment of RISs in the environment can allow effective manipulation of the THz signals, thus mitigate signal attenuation, and enhance communication coverage. With THz communication, V2X systems can transmit large amounts of data quickly and reliably, facilitating the real-time exchange of information critical for autonomous driving, collision avoidance, and traffic coordination. Moreover, THz communication enables precise localization and mapping, thereby enhancing the accuracy of V2X applications. By harnessing the potential of THz in V2X scenarios, we can envision a safer and more efficient transportation ecosystem.

### 5.2. Enhanced Precision in Location and Sensing for Platooning

In platooning, vehicles travel close together in a convoy, which improves fuel efficiency and reduces traffic congestion. However, platooning requires high-precision location and sensing to ensure that vehicles can maintain a safe distance from each other. An RIS can be used to improve the accuracy of location and sensing in platooning by reflecting signals in a manner that enhances the performance of V2X communication. Several challenges must be addressed to realize the full potential of RIS-aided V2X platooning, including the development of efficient algorithms for controlling RISs and developing RIS-aware V2X protocols.

### 5.3. AI-Enabled RIS-Aided V2X

AI has tremendous potential for RIS-aided V2X communications. Several critical challenges can be addressed by integrating AI algorithms into the RIS-empowered vehicular networks. AI can dynamically optimize the configuration of RIS elements by adapting to the changing environmental conditions and communication requirements in real time. This adaptability ensures that the RIS optimally reflects and refracts radio waves, thereby enhancing the signal strength and coverage. Leveraging data-driven deep learning techniques offers an opportunity to empower ITS and drive advancements in autonomous driving within vehicular networks. Moreover, an AI-driven RIS can efficiently mitigate signal interference and blockages, thus enhancing the reliability and robustness of V2X communication.

### 5.4. The Security of RIS-Aided V2X

The RIS-assisted V2X has a lot of potential to improve the security of V2X connections. To guarantee this technology’s safe and secure adoption, it is crucial to thoroughly examine and handle the security aspects of it. RIS-assisted V2X may be vulnerable to several kinds of security risks, including eavesdropping, denial-of-service (DoS) attacks, and man-in-the-middle assaults. Implementing strong security measures is essential to protect RIS-aided V2X. To safeguard communication routes, methods like encryption, authentication, and integrity checking are essential. The RIS-aided V2X can successfully mitigate security threats and provide a safer and more dependable vehicular communication environment by rigorously implementing these security measures.

## 6. Conclusions

In conclusion, academic research on reconfigurable intelligent surfaces and their incorporation into vehicle-to-everything communication has attracted a lot of attention. This article provides a comprehensive exploration of the opportunities presented by RIS in supporting V2X communication, drawing on a holistic survey of applications and emerging research activities in the field. Separate discussions on RIS and V2X research, along with the potential benefits and challenges associated with RISs in vehicular networks, are discussed. In addition, an examination of major RIS-aided V2X applications and future technologies sheds light on the potential of this integration. To bridge the gap between present and upcoming studies, the difficulties with combining RIS with V2X were recognized, and future study options were suggested. Research on RIS-V2X communication is still in its early stages, thus there is still a lot to explore and uncover in this area. With RIS poised to transform vehicular networks and enhance service quality and user experience, this timely work will inspire researchers and stakeholders to intensify their efforts in this promising area of research.

## Figures and Tables

**Table 1:** Existing surveys on RIS-V2X and our contributions.

Related Work	TOPIC	Key Contributions
[16]	5G NR V2X communication	This in-depth tutorial focuses on the 3GPP Release 16 5G NR V2X standard, particularly on the sidelink aspect.
[15,17]	6G for vehicular networks	The article analyses the advantages and drawbacks of LTE which has been explored and adopted as a wireless communication technology for vehicular communications and identifies essential enabling technologies from diverse domains, including new materials, algorithms, and system architectures.
[19,22]	Security and privacy in V2X	The paper discusses security and privacy aspects across the entire system stack, with a particular emphasis on road safety.
[20]	Cybersecurity mechanisms in V2X	A comprehensive examination of the current body of literature concerning V2X security is undertaken. Furthermore, the exploration extends to the possibility of integrating novel security methodologies that leverage Artificial Intelligence techniques to attain heightened security goals within V2X communications.
[23]	Hardware of RIS	A comprehensive review of RISs is presented, with a specific emphasis on the hardware aspect of RIS technology.
[24]	RIS-aided wireless communication	Survey provides a comprehensive overview of RIS in the context of wireless communications., addressing key issues by discussing reflection and channel models, hardware architecture, practical constraints, and diverse applications in wireless networks.
[28,29]	Potentials, applications, and challenges of RIS	This tutorial provides an overview of RIS in wireless communications and explores the potential performance improvements that arise from integrating RISs with emerging communication technologies.
[5]	Principles and opportunities of RIS	A comprehensive assessment of the latest advancements in RISs is conducted, encompassing areas such as operational principles, performance assessment, beamforming configuration, resource administration, and the integration of machine learning techniques in wireless networks enhanced by RIS technology.
[11]	Metasurfaces in vehicular networks	The main aim of this paper is to examine the limitations of existing wireless access technologies in vehicular scenarios and evaluate the potential impact and improvement that reconfigurable metasurfaces might have on future applications involving vehicles.
[30]	RIS-aided vehicular networks	In this brief overview, effective transmission methods to tackle the difficulties arising from RIS-assisted V2X communications are introduced.
[32]	Intelligent and secure radio environments for 6G vehicular aided HetNets	The authors present a design approach aimed at enhancing the efficiency of 6G vehicular-assisted HetNets, resulting in improved reliability, security, and energy efficiency. Their methodology involves the integration of deep learning and reconfigurable metasurfaces, offering a cost-effective alternative in comparison to conventional phased array antennas for disrupting eavesdropper reception
Our Work	Vehicular networks with RIS: state-of-the-art review	A comprehensive survey focusing on the enhancement of V2X communication through RIS, particularly, We highlight the potential benefits and challenges of this combination. We examine several use cases of RIS-aided V2X communication, namely extended coverage, spectrum sharing, resource allocation, platooning, physical layer security and autonomous vehicles. We delve into the fundamental emerging technologies that are anticipated to empower vehicular networks, encompassing MEC, NOMA, mmWave communication, Artificial Intelligence, and Li-Fi. Classified tables are provided for the use case and emerging technologies. Research challenges and potential future direction are also emphasized.

**Table 2:** List of key acronyms.

Acronyms	Definitions
RIS	Reconfigurable Intelligent Surface
V2X	Vehicle to Everything
MEC	Mobile Edge Computing
NOMA	Non-Orthogonal Multiple Access
AI	Artificial Intelligence
VLC	Visible Light Communication
IoV	Internet of Vehicle
SNR	Signal-to-Noise Ratio
CAV	Connected Autonomous Vehicle
ITS	Intelligent Transportation System
SWIPT	Simultaneous Wireless Information and Power Transfer
VRU	Vulnerable Road User
PLS	Physical Layer Security
NS	Network Slicing
D2D	Device-to-Device
CSI	Channel State Information
LOS	Line of Sight
NLOS	Non-Line of sight
QoS	Quality of Service
SOP	Secrecy Outage Probability
THz	Terahertz
RSU	Roadside Unit
DRL	Deep Reinforcement Learning
VANET	Vehicular Ad hoc Network
LiDAR	Light Detection and Ranging
RF	Radio Frequency

**Table 3:** Comparison table of DSRC-based V2X and LTE-based V2X.

Feature	DSRC-Based V2X	LTE-Based V2X
Released	2010	2016
Frequency band	5.9 GHz	Cellular network
Range	Good for short radio range	Good for extended communication
Reliability	Good	Excellent
Modulation	OFDM	SC-FDM
Latency	Low for V2V	Low
Devices	Available	Not yet available
Deployment status	Deployed in some countries	Deploying in several countries
Security and privacy	N/A	Yes

**Table 4:** Benefits and challenges associated in RIS-aided V2X.

RIS-AIDED V2X	
Benefits	Challenges
Range Extension: RIS can mitigate NLOS issues by redirecting and reflecting the signals to reach the receiver through alternative paths.	Double Fading: This effect leads to significantly larger path losses compared to the direct link, making it challenging for passive RISs to achieve substantial capacity gains in many wireless environments
Energy Efficient: RIS can be used to focus radio waves on a specific direction, which can reduce the amount of power needed to transmit the signal.	High Path Loss: The specific design and configuration of the RIS, as well as the signal frequency and incident angles, can affect the efficiency of signal reflection and the resulting path loss.
Increased Reliability: Improving the reliability of wireless communication systems, including those assisted by RIS, can be achieved through these measures optimizing signal reception, reducing interference, extending the communication range, and enhancing security.	Cost and Infrastructure: The costs associated with manufacturing, installing, and maintaining many RIS elements throughout a vehicular network can pose practical and financial challenges for deployment.
Security: By controlling the reflection of radio waves, RIS can be used to create one-way or two-way channels that are only accessible to authorized vehicles. This can help to prevent eavesdropping and spoofing attacks.	Channel Estimation: The performance of RIS-aided V2X systems is affected by the Doppler-induced channel aging effect. This effect deteriorates the system’s performance, particularly in terms of resource allocation. The success of resource allocation heavily relies on accurate and timely CSI.

**Table 5:** Classification of RIS-aided V2X applications.

Applications	Ref.	Optimization Techniques	Key Contributions	Limitations
Extended Coverage	[10]	LIMoSim	The possible advantages and challenges associated with implementing RIS to enhance mmWave networks and amplify coverage capabilities in vehicular application scenarios have been showcased.	The effectiveness of reflecting surfaces may be affected by these the positions and orientations of vehicles, requiring efficient algorithms and mechanisms for adapting and adjusting the reflecting surfaces in real time.
	[54]	Monte Carlo	The outage probability in vehicular communication systems assisted by RIS is studied.	The efficient deployment and control of UAVs in real-world scenarios need to be addressed, considering factors such as airspace regulations, energy constraints, and coordination with ground infrastructure.
	[55]	DRL and block coordinate descent (BCD)	The core emphasis of this study centers on a system model that employs RIS to elevate wireless connectivity for vehicles traversing areas with limited signal coverage.	Compliance with regulations, airspace management, and ensuring the safe operation of IRS-equipped systems are important considerations that need to be addressed.
	[56]	Sweeping algorithm	This paper presents a compelling approach that harnesses RIS to establish virtual line-of-sight (VLoS) paths.	These models may not fully capture the complexity of real-world wireless channels, which can be influenced by factors such as multipath fading, shadowing, and NLOS conditions.
Resource Allocation	[60]	Link selection algorithm	In this paper, a resource allocation strategy is presented with the aim of improving dependability and diminishing latency within VANETs.	The scalability of the proposed resource allocation scheme is an important aspect that may require further investigation.
	[61]	DRL-based algorithm	To achieve the dual objectives of maximizing the sum capacity for vehicle-to-infrastructure users and satisfying the latency and reliability requirements of vehicle-to-vehicle pairs.	The computational resources and time needed for training should be considered, especially in dynamic and time constrained V2X scenarios.
	[1]	RAIVC algorithm	Elevating the quality of service (QoS) within vehicular communication frameworks is explored.	Acquiring CSI in vehicular scenarios can be challenging due to fast-changing channel conditions, mobility, and dynamic obstacles.
	[30]	Three-layer DTS framework	An original and unique frame architecture was devised explicitly for the purpose of enhancing V2X communications with the assistance of RIS. The aim was to decrease the overhead related to signaling.	Practical deployment challenges, such as the cost of deploying RIS elements, the availability and placement of RIS in real-world vehicular environments, and the coordination with existing infrastructure, could be addressed.
Spectrum Sharing	[69]	Gradient-based linearization domain algorithm	This piece offers a comprehensive survey of spectrum-sharing systems (SSS) empowered by RIS, underscoring the conceivable advantages.	The practical spectrum policy considerations and regulatory aspects related to RIS-aided spectrum sharing can be highlighted.
	[70]	JRARO algorithm	This study investigates the collective optimization issue in V2X networks enhanced by RIS. The main emphasis lies on examining how the quality of the channel and the mutual trust among V2X links are interconnected.	The trade-off between optimizing social metrics (e.g., fairness, social equity) and traditional network performance metrics (e.g., throughput, latency) could be addressed.
	[71]	AOIA algorithm	To fulfill quality of service (QoS) demands within V2X communications, this research simultaneously fine-tunes several parameters.	The integration of RISs into higher-layer protocols and the impact of RIS deployment on network-level performance metrics can be investigated.
Physical Layer Security	[74]	Element-wise BCD and Ao-MM algorithm	An innovative method for augmenting the security of wireless communication networks at the physical layer is presented, involving the incorporation of IRSs.	Further experimental evaluation or implementation of the proposed intelligent V2X security (IV2XS) framework is required.
	[14]	Monte Carlo	This study introduces an original examination of the PLS in vehicular networks aided by RIS. The efficacy of the suggested systems is evaluated using criteria such as the average secrecy capacity (ASC) and the secrecy outage probability (SOP).	An extensive discussion or analysis of the practical implementation challenges and feasibility of deploying RISs in vehicular networks can be provided.
Autonomous Vehicle	[4]	Optimum RIS positioning algorithm	In this paper, a structural remedy is suggested to amplify the dependability of autonomous vehicular networks.	It lacks extensive experimental validation or field trials to demonstrate the practical feasibility and performance of the proposed RIS-supported architecture in real-world scenarios.
	[75]	CG-HB and double-step iterative algorithm	To address the challenge of blockage awareness in autonomous vehicles, a new RIS-assisted mmWave MIMO channel model is proposed.	The effectiveness and practical feasibility of implementing these PLS techniques in real V2X networks may require further investigation and validation.
	[76]	CoopeRIS	This paper investigates the integration of cooperative driving systems and RIS-enabled mmWave communications.	Investigating the impact of imperfect CSI on the system performance and developing robust techniques to mitigate the effects of CSI inaccuracies could be focused.
Platooning	[80]	DP-SAPC algorithm	The intention is to improve the effectiveness of this setup through a dual objective: firstly, augmenting the quantity of vehicles in each platoon, and secondly, minimizing energy consumption.	The lack of analysis regarding the cost and practical feasibility of implementing the proposed RIS-assisted algorithms.
	[81]	PLEXE framework	The authors suggested the implementation of RISs to facilitate and enable cooperative driving maneuvers in urban scenarios in the future.	A comprehensive evaluation of the potential security vulnerabilities and robustness of the proposed system could add more value in RIS-enabled cooperative driving.
	[82]	DiLuS -STPL framework	The proposed algorithm utilizing RISs has the potential to effectively address the misleading effects caused by NLoS channels and fluctuations in the number of NLoS paths.	An in-depth analysis of the system’s robustness under various real-world conditions and scenarios could be provided.

**Table 6:** Taxonomy of emerging technologies for RIS-aided vehicular networks.

Emerging Technology	Key Features	Potential Application in RIS-Aided V2X
Mobile Edge Computing	It is a distributed computing paradigm that brings computational resources and services closer to the edge of the network, typically at the base stations or access points. It enables data processing, storage, and computing capabilities at the edge of the network, reducing latency and enhancing the overall performance of applications and services for end-users. MEC integration in V2X has the potential to enhance overall network performance, reduce latency, support real-time applications, and improve the efficiency and reliability of communications between vehicles and infrastructure, fostering safer and more efficient transportation systems.	A novel scheme that synergizes RIS with edge computing is proposed in [88], to reduce overall task offloading latency. The proposed approach utilizes an actor-critic DRL algorithm to optimize the task offloading process, effectively leveraging the benefits of both RIS and edge computing to enhance system performance and minimize latency. The article [89] focuses on the study of MEC-enabled vehicular networks, leveraging the assistance of IRS to enhance the computing performance of the system. To address the limitations posed by the availability of IRS links and MEC processors, the task scheduling is optimized through an analysis of processor resource allocation and offloading policies.
Non-Orthogonal Multiple Access	It is a multiple access technique used in wireless communication systems, where multiple users can share the same frequency and time resources simultaneously. Unlike traditional orthogonal multiple access (OMA) techniques, NOMA allows multiple users to use the same resources simultaneously by applying power domain multiplexing. NOMA’s capability to handle asymmetric channel conditions and accommodate a dynamic number of active users makes it a suitable candidate for V2X scenarios, where vehicle density and communication demands can vary rapidly. It has the potential to improve spectral efficiency and overall performance, contributing to safer and more efficient V2X systems.	A novel approach called simultaneously transmitting/refracting and reflecting reconfigurable intelligent surface (STAR-RIS) NOMA to achieve comprehensive coverage in particular area is proposed in [96]. In this study, approximate expressions for the outage probability of user n using either inter-user power successive interference cancellation (ipSIC) or partial successive interference cancellation (pSIC) is derived, as well as for user m, in the presence of Rician fading channels. To address this issue, a NOMA-based IoV network assisted by RIS is proposed in [97].
mmWave/THz Communications	mmWave communication operates in the frequency range from 30 GHz to 300 GHz while THz communication operates at even higher frequencies, typically ranging from 300 GHz to 10 THz. These extremely high frequencies provide even larger bandwidth, promising data rates in the multi-gigabit-per-second range and beyond. The integration of these technologies into V2X networks can contribute to safer, more efficient, and connected transportation systems, empowering the realization of intelligent and autonomous driving in the future.	The integration of mmWave communication is expected to play a critical role in supporting the advanced use cases of automated driving. High-resolution and real-time maps, known as dynamic High definition (HD) 3D maps, are essential for the safe navigating of vehicles in automated driving systems [100]. In [102], a risk-aware learning-based framework with the DRL approach is introduced. The framework aims to enhance road safety, which is a primary objective of ITSs. A simulation framework called LIMoSim (Lightweight ICT-centric Mobility Simulation) has been developed to effectively explore the possibilities of utilizing RIS for optimizing coverage in vehicular networks in [10]. Initial findings demonstrate the potential of RIS deployment in enhancing coverage for a specific vehicular application use case.
Artificial Intelligence	AI is a transformative force in V2X communications, enabling smarter transportation systems. With AI’s capabilities, V2X can optimize traffic management, power autonomous driving systems, enhance V2X communication by dynamically allocating resources, predict maintenance needs, enable natural language interaction with vehicles, and optimize smart infrastructure. By leveraging AI’s potential, V2X can achieve safer, more efficient, and connected mobility, revolutionizing the driving experience, and laying the foundation for the future of intelligent transportation.	Numerous research studies have been carried out on the implementation of machine learning in RIS-assisted V2X communications. In [108] The authors addressed the power allocation issue in the V2X environment by formulating it as the Weighted Minimum Mean Square Error (WMMSE) problem. The authors in [109] introduced a novel channel estimation method for an IRS-assisted B5G/6G network utilizing supervised learning based on Convolutional Neural Networks (CNNs). They considered the transmission power of the signal and aimed to minimize power consumption. The proposed framework in [110] introduces a hybrid vehicular communication structure combining intelligent reflecting surface (IRS) and DSRC-cellular technology. An AI-empowered communication framework is presented for VANETs, aiming to achieve energy efficiency, high data rates, and low-latency transmission. A control scheme called D-PPC (Deep learning-based IRS Phase Shift and Power Allocation Control), which utilizes deep learning techniques to optimize the phase shift and power allocation in an IRS is proposed in [111].
Visible Light Communication	VLC is an emerging technology that utilizes light signals, typically from LEDs (Light-Emitting Diodes), to enable data transmission between vehicles and infrastructure. VLC can be used for a variety of V2X applications, such as collision avoidance, traffic management, and parking assistance. VLC offers higher data rates, lower latency, and more security than traditional wireless technologies, making it a potential game-changer for V2X communication.	Deploying RIS arrays to facilitate communication between vehicles that are moving parallel to each other was suggested in [113], since VLC transceivers have a limited field of view (FOV), maintaining alignment between two vehicles traveling beside each other becomes difficult. A stochastic geometry-based analytical framework to assess the performance of our proposed solutions concerning outage probability, throughput, and delay outage rate (DOR) was introduced in [3], to address higher interference, reduced packet reception rate, and longer communication delays due to channel congestion in vehicular-radio frequency V-RF. The study in [114] introduces a VLC communication infrastructure designed specifically for vehicles, considering the unique characteristics of road tunnels, while researchers in [115] explore how reconfigurable intelligent surface (RIS) technology can effectively reduce the blocking probability (BP) within tunnels and mitigate signal blockage issues.
